# The Effect of Processing Methods and Nucleating Agents on the Wear Resistance and Crystallinity Behavior of Nylon 11

**DOI:** 10.3390/polym17081073

**Published:** 2025-04-16

**Authors:** Hu Lyu, Dongzhou Sun, Yue Li, Guoliang Yu, Shudi Liu, Pengfei Huo, Dawei Zhang, Xianzhi Kong

**Affiliations:** 1 Institute of Petrochemistry, Heilongjiang Academy of Sciences, Harbin 150040, China; haitun@hipc.org.cn (H.L.); sundongzhou@hipc.org.cn (D.S.); liyue@hipc.org.cn (Y.L.); yuguoliang@hipc.org.cn (G.Y.); liushudi@hipc.org.cn (S.L.); 2 Engineering Research Center of Advanced Wooden Materials (Ministry of Education), Northeast Forestry University, Harbin 150040, China; huopengfei@nefu.edu.cn

**Keywords:** crystallization, melting point, nylon 11, polymer processing, nucleation

## Abstract

Nylon 11 is widely used in abrasion-resistant coatings due to its excellent wear resistance and processability. Here, the effects of different processing methods (pre-treatment temperatures, melting temperatures, and heating programs) and nucleating agents (silica, talcum powder, and montmorillonite) on the crystallinity behavior and wear resistance of Nylon 11 were systematically analyzed. The results show that pre-treating Nylon 11 at 80–100 °C enhances its wear resistance, and its friction coefficient drops to ~0.16. Melting temperature influences both the processing flowability and wear resistance of Nylon 11. Specifically, when the melting temperature exceeds 195 °C, wear resistance improves significantly and its friction coefficient decreases from 0.32 to 0.17. Moreover, variations in the heating program also affect the wear resistance of Nylon 11. Optimal wear resistance is achieved when Nylon 11 is held at both 165 °C and 185 °C for 10 min (friction coefficient: ~0.17). The nucleating agents (silica, talcum powder, and montmorillonite) do not change the crystalline morphology of Nylon 11, which predominantly exhibits an orthorhombic α-phase. However, as the content of nucleating agents increases (0–1 wt%), the crystallinity first rises and then declines, with its highest value being 46.48%. This work emphasizes the critical role of processing methods and nucleating agents in the wear resistance and crystallinity behavior of Nylon 11, providing valuable insights for their performance optimization.

## 1. Introduction

Nylon is a widely utilized polymer known for its excellent mechanical properties and thermal stability [1,2]. High crystallinity in Nylon contributes to enhanced tensile strength, rigidity, and thermal resistance, making it suitable for various engineering applications [3]. The processing methods employed in the manufacturing of Nylon significantly influence its crystallization behavior, with factors such as cooling rate, melting temperature, and processing time affecting the crystalline morphology [4,5,6]. For instance, a slow cooling rate during melt processing tends to favor the formation of larger crystalline domains, while rapid cooling can result in a more amorphous structure [6,7].

The incorporation of nucleating agents and other processing additives also plays a critical role in modulating the crystallization performance of Nylon [8,9,10]. Nucleating agents serve as effective sites for crystal growth, thereby enhancing the crystallization rate and degree of crystallinity [11,12]. Various studies have shown that the choice of nucleating agents, such as talcum powder [13], silica [14], or montmorillonite [15], can lead to distinct variations in crystallization temperatures and morphological characteristics. Furthermore, the distribution and concentration of these nucleating agents within the Nylon matrix are imperative for achieving uniform properties and improving thermal and mechanical performance [9,16]

Wear resistance is also a critical property of Nylon, particularly in applications where friction and wear are of paramount importance [17,18,19]. The frictional performance of Nylon is also closely linked to its crystallinity; generally, higher crystallinity correlates with reduced friction coefficients and improved wear resistance [18,20]. Nylon materials with a high degree of crystallinity have more regular and orderly arrangements of molecular chains, resulting in a smoother and flatter surface. Moreover, due to the close arrangement of molecular chains, the intermolecular forces are relatively strong, forming a more stable crystal structure. Therefore, their wear resistance is better.

Nylon 11, a member of the Nylon family, is widely used in abrasion-resistant coatings due to its excellent wear resistance and processability, and has garnered significant attention in materials science and engineering due to its bio-based nature which aligns with global sustainability initiatives. With the growing applications of Nylon 11 in mechanical components, understanding the relationships between crystallization behavior, processing methods, and tribological performance is integral to advancing the development of Nylon materials tailored for high-performance applications. Here, a detailed comparative analysis of the effect of various processing methods (pre-treatment temperatures, melting temperatures, and heating programs) and nucleating agents (silica, talcum powder, and montmorillonite) on the crystallinity and wear resistance of Nylon 11 was conducted.

## 2. Materials and Methods

### 2.1. Materials

Nylon 11 (size: 80–90 μm, Mn: 12,900 Da, Mw: 30,836 Da, purity: ≥99.9%,) was purchased from Shanghai Xinhao Chemical Co., Ltd. (Shanghai, China). Silica (purity: ≥90%), talcum powder (size: 10 μm), and montmorillonite (K-10) were purchased from Shanghai Tengzhun Biotechnology Co., Ltd. (Shanghai, China).

### 2.2. The Processing Methods

The different pre-treatment temperatures. Multiple samples of 6 g Nylon 11 were taken and placed in aluminum foil trays measuring 5 × 5 × 3 cm. The Nylon 11 samples were flattened and subjected to a 1 h pre-treatment in an air-circulating oven with varying temperatures set at 60 °C, 80 °C, 100 °C, 120 °C, 140 °C, 160 °C, and 180 °C. Subsequently, 4 g of pre-treated Nylon material was placed in the hopper of a micro-injection molding machine and pre-heated to melt for 150 s. The injection pressure was set at 0.5 MPa, and the dimensions of the molded specimen was 30 mm × 7 mm × 6 mm, designated for wear resistance testing.

The different melting temperatures. The Nylon 11 was dried in an air-circulating oven at 80 °C for 1 h. Following this, 4 g of the dried Nylon 11 powder was placed into the barrel of a micro-injection molding machine and melted for 150 s. The barrel temperatures were set at 190 °C, 195 °C, 200 °C, 205 °C, and 210 °C. The mold used was selected according to the specifications outlined in GB/T 1040-18 (Plastics–Determination of tensile properties–Part 1: Genaranl principles), and its temperature was set to 50 °C.

The different heating programs. Heating program 1: Nylon 11 was dried in an air-circulating oven at 80 °C for 1 h. Subsequently, 4 g of the dried Nylon 11 powder was placed into the barrel of a micro-injection molding machine. The barrel temperature was maintained at 165 °C for 10 min before being elevated to 195 °C. Once the temperature reached 195 °C, timing commenced, with a melting duration of 150 s, while the mold temperature was set to 50 °C; Heating program 2: Nylon 11 was dried in an air-circulating oven at 80 °C for 1 h. Subsequently, 4 g of the dried Nylon 11 was placed into the barrel of a micro-injection molding machine. The barrel was maintained at 165 °C for 10 min, after which the temperature was increased to 185 °C and held for an additional 10 min. The barrel temperature was then raised to 195 °C. Once the temperature reached 195 °C, timing commenced, with a melting duration of 150 s, while the mold temperature was set to 50 °C; Heating program 3: Nylon 11 was dried in an air-circulating oven at 80 °C for 1 h. Subsequently, 4 g of the dried Nylon 11 was placed into the barrel of a micro-injection molding machine. The barrel temperature was maintained at 165 °C for 10 min, after which it was raised to 175 °C and held for an additional 10 min. The barrel temperature was then increased to 185 °C and maintained for another 10 min before finally being elevated to 195 °C. Once the temperature reached 195 °C, timing commenced, with a melting duration of 150 s, while the mold temperature was set to 50 °C.

### 2.3. The Nucleating Methods

Nylon 11 was placed in an electric air-circulating oven at 80 °C for 12 h. Following this, the Nylon 11 was mixed uniformly with nucleating agents (silica, talcum powder, and montmorillonite). The mixture was then extruded using a screw extruder at a rotation speed of 65 rpm to 90 rpm. The extrudate was subsequently crushed, and the resulting granules were dried in an oven at 80 °C for 8 h to obtain Nylon granules, which were reserved for further use.

### 2.4. Characterization and Measurements

Fourier-transform infrared spectroscopy analysis (FTIR). The FTIR of Nylon 11 samples subjected to different temperature treatments for 1 h were performed on a TENSORII (Bruker, Berlin, Germany) collecting 32 scans (resolution: 4 cm^−1^) from 400 to 4000 cm^−1^.

Differential scanning calorimetry testing (DSC). The degree of crystallinity of the samples was determined by analyzing their DSC curves. DSC (DSC214, NETZSCH, Selb Germany) was utilized to measure the melting temperature (T_m_) and the melting enthalpy (Δ*H_f_*) of Nylon 11 and its composite materials, to calculate the material’s crystallinity (*X_c_*). Following sample preparation, the heating rate was set at 5 °C/min up to 230 °C, holding for 5 min; subsequently, the material was cooled at a rate of 5 °C/min to 30 °C, then held for 5 min, followed by reheating to 230 °C at 5 °C/min and holding for 5 min, and finally cooling to room temperature at 5 °C/min. The relative crystallinity was calculated according to Equation (1):

(1) $$X_{c}=\frac{\Delta H_{f}}{\phi \times \Delta H_{m}^{0}}\times 100\%$$

where Δ*H_f_* represents the sample’s melting enthalpy (J/mol); Δ$H_{m}^{0}$ denotes the melting enthalpy of fully crystallized Nylon 11 (Δ$H_{m}^{0}$ = 189 J/g); and *∅* represents the mass fraction of Nylon 11 in the composite material.

X-ray diffraction analysis (XRD). XRD was conducted to observe the crystalline structure of the samples. This was performed on the Nylon 11 samples using a D8 ADVANCE (Bruker, Berlin, Germany). Cu Kα radiation was employed as the radiation source, with a testing voltage of 30 kV and a current of 10 mA. The scanning range was set from 5° to 60° with a scan speed of 0.09 rad/s.

Wear resistance performance. The rectangular specimens prepared by injection molding were secured in a plastic friction and wear testing machine with an experimental load of 20 kg. Using a grinding disc with a radius of 20 mm rotating at a speed of 200 rpm, the dimensions, width, thickness, density, and initial mass of the rectangular specimens were input. The testing commenced and continued until abnormal noise was detected in the plastic friction and wear testing machine or until the test automatically ended. Subsequently, the rectangular specimens were removed, and weighed, and the wear width was measured to determine the coefficient of friction.

Softening point test. The Vicat softening point was determined using a Vicat softening temperature tester for heat deformation. The heating rate was set at 50 °C/h within a temperature range of 25 to 200 °C. The temperature at which a cylindrical sample with a cross-sectional area of 1 mm^2^ vertically indented by 1 mm under a 5 kg load represents the heat deformation temperature.

## 3. Results and Discussion

### 3.1. The Effect of the Pre-Treatment Temperature on the Crystallinity and Wear Resistance

**Figure 1:**
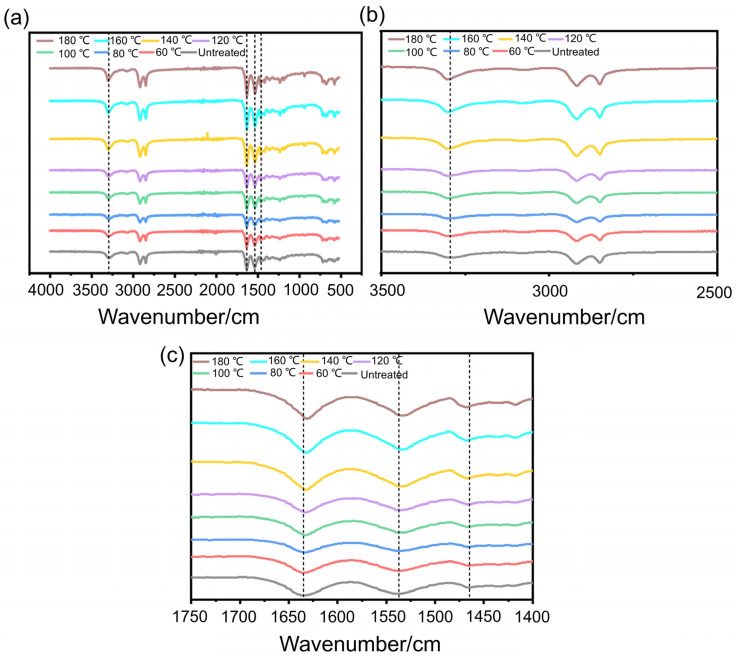
FTIR spectra of Nylon 11 at different pre-treatment temperatures. (**a**) FTIR spectra, (**b**) FTIR spectra between 2500–3000 cm^−1^, (**c**) FTIR spectra between 1400–1750 cm^−1^.

To investigate the impact of pre-treatment temperature on the chemical structure of Nylon 11, its infrared spectroscopy was conducted. Figure 1a shows the infrared spectra of Nylon 11 at different processing temperatures. It can be observed that the peaks in the range of 640 cm^−1^ to 1540 cm^−1^ correspond to the stretching vibrations of the amide I and II bands [21], while the peak at 1462 cm^−1^ represents the stretching vibration of the -CH_2_- bonds in the diamine segments. The stretching vibration peak of NH is located at 3297 cm^−1^ [22], and with increasing processing temperature, the NH bond in Nylon 11 shows a slight shift, with the peak shape becoming progressively sharper, as shown in Figure 1b. Meanwhile, the characteristic peak of C=O in Nylon 11 is located around 1633 cm^−1^ [23], and it is evident that after high-temperature treatment, the peak shifts, which may be attributed to the formation of hydrogen bonding interactions between the molecular chains of Nylon 11, as depicted in Figure 1c. This indicates that the effect of water molecules on the hydrogen bonds between Nylon 11 molecules changes with varying pre-treatment temperatures.

**Figure 2:**
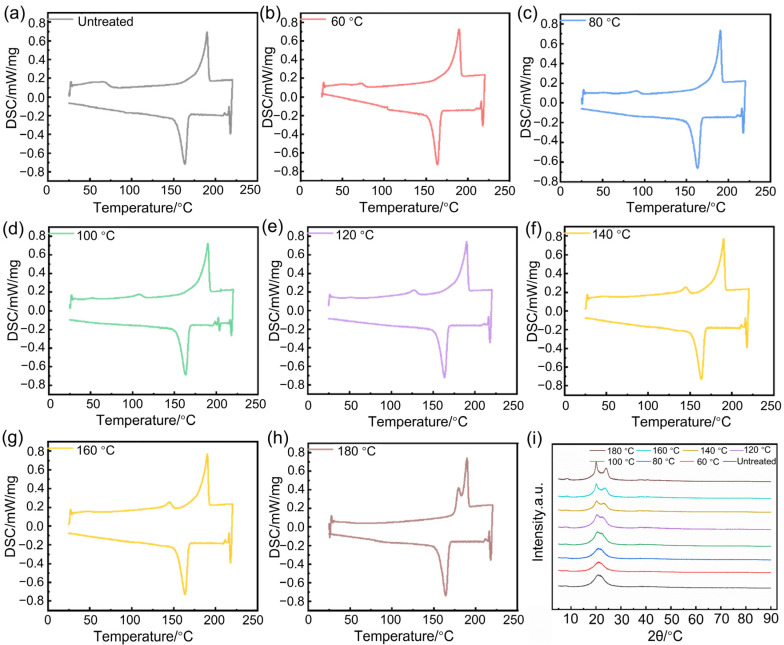
DSC and XRD curves of Nylon 11 at different pre-treatment temperatures. (**a**) Untreated, (**b**) 60 °C, (**c**) 80 °C, (**d**) 100 °C, (**e**) 120 °C, (**f**) 140 °C, (**g**) 160 °C, (**h**) 180 °C, (**i**) XRD curves.

DSC analysis was performed on Nylon 11 subjected to different pre-treatment temperatures. As shown in Figure 2a–h, there is no apparent glass transition temperature (*T_g_*) observed during the heating process for Nylon 11 pre-treated at various temperatures. This may be attributed to the rapid formation of hydrogen bonding through the N-H bonds in Nylon 11, which accelerates the crystallization process, resulting in a relatively complete crystallization. The melting of Nylon 11 is characterized by a bimodal peak. This was attributed to that the melting peak at lower temperatures corresponds to the oriented segments of the Nylon 11 macromolecular chains, while the higher temperature melting peak occurs at 190 °C, representing the melting peak of Nylon 11 [24]. The crystallization temperature of Nylon 11 is relatively high (163 °C), and the sharp and narrow crystallization peak further indicates its rapid crystallization rate. As the heat treatment temperature increases, the water content in Nylon 11 gradually evaporates, reducing the “plasticizing” effect and leading to a migration of the low-temperature melting peak toward higher temperatures.

After subjecting Nylon 11 to drying at different temperatures for 1 h, the water molecules within the Nylon 11 begin to evaporate. As the pre-treatment temperature increases, the melting point of Nylon 11 exhibits little variation, while both the melting enthalpy and crystallinity display a trend of initially decreasing before subsequently increasing. Table 1 summarizes the melting point, melting enthalpy, and crystallinity of Nylon 11 at different processing temperatures. Notably, the minimum melting enthalpy and crystallinity occur when Nylon 11 is treated at 80 °C for 1 h, resulting in optimal melt fluidity. At this temperature, the water molecules in Nylon 11 are activated, disrupting the hydrogen bonding interactions between the Nylon 11 molecules, which leads to a reduction in crystallinity and melting enthalpy, thus facilitating the flow of Nylon 11. As the temperature continues to rise, the water molecules gradually evaporate, and the crystallinity of Nylon 11 approaches its inherent level.

Figure 2i depicts the X-ray diffraction (XRD) patterns of Nylon 11 composites treated at different pre-treatment temperatures. The materials exhibit the characteristic α-crystalline form, with the (100) crystalline plane appearing around 2θ = 20° and the (010)/(110) crystalline planes observed near 2θ = 24°. However, at lower processing temperatures (room temperature to 80 °C), no distinct crystalline peaks are evident, likely due to the presence of water molecules disrupting the crystallinity of the Nylon 11 molecular chains. As the pre-treatment temperature gradually increases to 100 °C, the emergence of dual peaks indicates that water molecules are gradually evaporating, leading to a more organized arrangement of the molecular chains and a subsequent enhancement in crystallinity. The XRD analysis results further confirmed that water molecules compete for the hydrogen bonds between Nylon 11 molecules.

**Figure 3:**
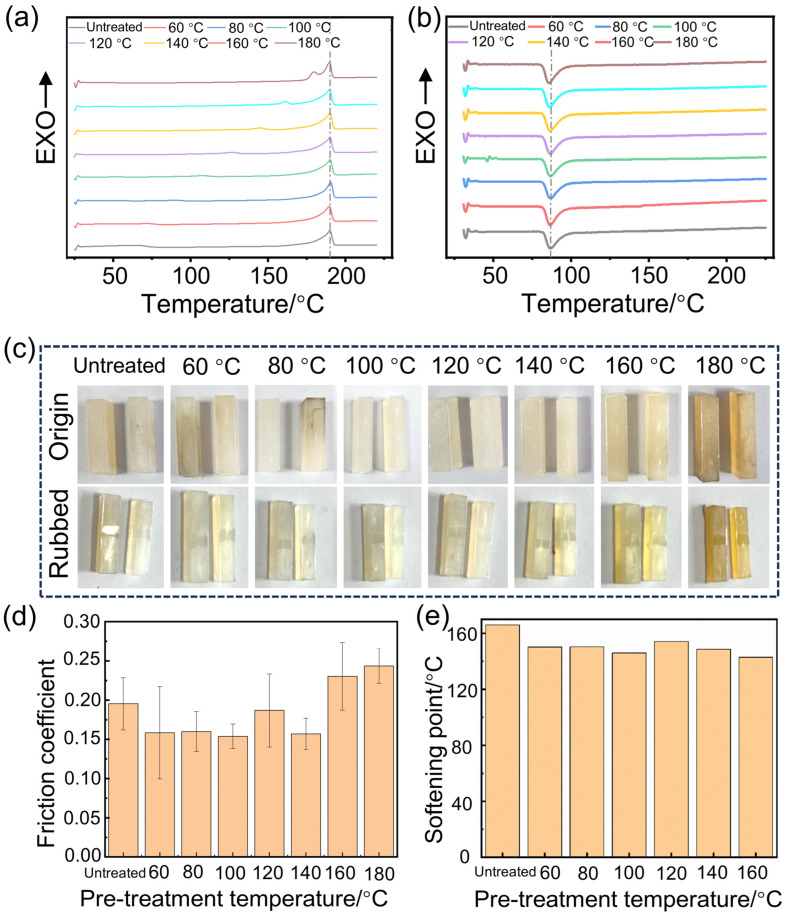
Heating and cooling DSC curves, original and rubbed digital photos, friction coefficients, and softening points of Nylon 11 at different pre-treatment temperatures. (**a**) Heating curves, (**b**) cooling curves, (**c**) the original and rubbed digital photos, (**d**) friction coefficients, (**e**) softening points.

Figure 3a,b illustrate the thermal behavior of pre-treated Nylon 11 during the heating process and its crystallization behavior upon cooling. During heating, the melting peak around 190 °C shows no significant shift, indicating that thermal treatment has minimal impact on the melting temperature, as seen in Figure 3a. However, in the cooling curves, it can be observed that as the heat treatment temperature increases, the water content in Nylon 11 gradually evaporates, reducing the “plasticizing” effect, as depicted in Figure 3b. This leads to a gradual migration of the low-temperature melting peak toward higher temperatures.

To investigate the impact of different pre-treatment temperatures on the wear resistance of Nylon 11, samples pre-treated at varying temperatures were injection molded into standard specimens for wear testing using an injection molding machine. To minimize the effect of heat generated during friction on the analysis of the structural influence of Nylon, the friction and wear tests were terminated after 40 min. Figure 3c presents digital photos of the origin and rubbed Nylon 11. Following the friction process, the wear amount remains relatively small, and the wear marks are not pronounced, indicating that Nylon 11 demonstrates excellent wear resistance. According to Figure 3d depicting the friction coefficients of Nylon samples subjected to different processing temperatures, it can be observed that the friction coefficient initially decreases and then increases with rising pre-treatment temperature. In the temperature range of 60 °C to 100 °C, the friction coefficients exhibit minimal variation (friction coefficient: ~0.16) and remain lower than those observed at higher temperatures, indicating superior wear resistance. This phenomenon may be attributed to the activation effect of water molecules within the system during processing at lower temperatures, which enhances the mobility of the molecular chains during molding. This improvement facilitates injection molding, resulting in a smoother and denser surface, thereby enhancing wear resistance. Consequently, this allows Nylon 11 during the spraying process to exhibit better flow properties, yielding a smoother surface with notably improved wear resistance.

Figure 3e illustrates the softening points of the samples at different pre-treatment temperatures. As the processing temperature increases, the softening point of Nylon 11 initially decreases; however, it subsequently rises when the temperature exceeds 100 °C. This behavior may be attributed to the activation of water molecules within the Nylon 11 matrix at temperatures below 100 °C, where the water remains within the system, enhancing the activation of Hydrogen bonding and resulting in a reduction of the softening temperature. Conversely, once the temperature surpasses 100 °C, the water molecules begin to evaporate, leading to a slight increase in the softening point. The trend in softening point variation closely parallels that of the friction coefficient.

### 3.2. The Effect of the Different Melting Temperatures on the Crystallinity and Wear Resistance

**Figure 4:**
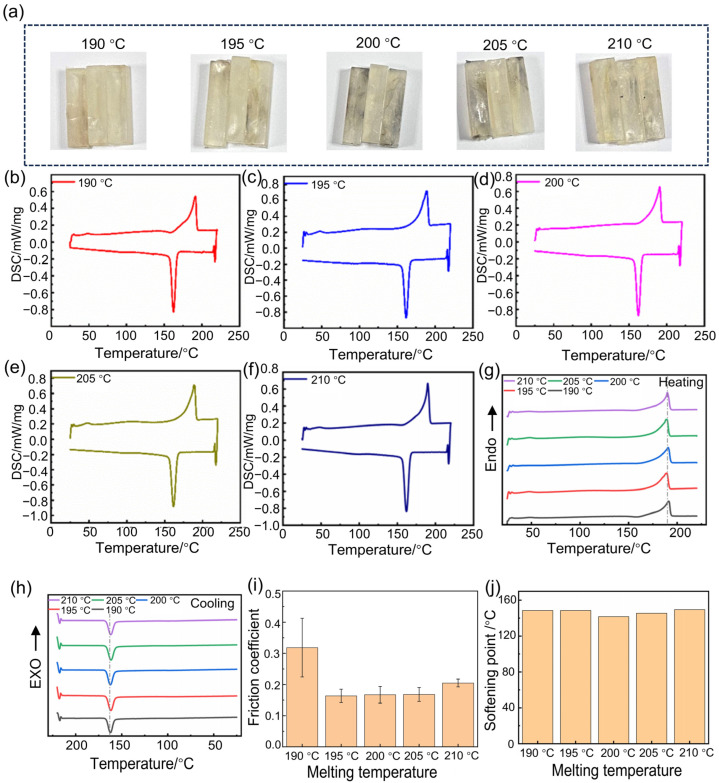
Effect of different melting temperatures on the crystalline and wear resistance of Nylon 11. (**a**) Digital photos, (**b**–**f**) DSC curves of (**b**) 190 °C, (**c**) 195 °C, (**d**) 200 °C, (**e**) 205 °C, (**f**) 210 °C, (**g**) heating curves, (**h**) cooling curves, (**i**) friction coefficient, (**j**) softening point.

The digital photos of wear-resistant specimens of Nylon 11 subjected to different melting temperatures were presented in Figure 4a. Figure 4b–f display the DSC curves of Nylon 11 samples prepared at different melting temperatures. During the heating process, the DSC curves exhibited no discernible glass transition, and the melting process was sharp and narrow, indicating that Nylon 11 retains good crystallinity after being subjected to different melting temperature treatments. Furthermore, distinct crystallization peaks were observed during the cooling process, demonstrating that Nylon 11 is capable of exhibiting significant crystallization behavior even after processing at various melting temperatures. The DSC curves for both the heating and cooling processes are summarized in Figure 4g,h, respectively. The melting temperatures of Nylon 11 treated at different melting temperatures are largely consistent, with similar peak shapes. Additionally, the cooling process curves distinctly exhibit sharp crystallization peaks, with the crystallization temperature approximately 163 °C, indicating favorable crystallization behavior. The melting point, melting enthalpy, and crystallinity of Nylon 11 at different melting temperatures are summarized in Table 2. The crystallinity of Nylon 11 is approximately 32%, showing no significant variation with increasing melting temperatures.

As indicated by Figure 4i, the friction coefficient of Nylon 11 is also influenced by melting temperature. The coefficient of friction is relatively high at 190 °C, while it reaches a minimum at 195 °C and decreases from 0.32 to 0.17, indicating optimal wear resistance at this temperature. Figure 4j illustrates the softening points of Nylon 11 samples at different melting temperatures. The softening point exhibits a trend of initially decreasing followed by an increase with rising melting temperatures. However, the changes are not particularly pronounced. Notably, the lowest softening point is observed at a melting temperature of 200 °C.

### 3.3. The Effect of the Different Heating Programs on the Crystallinity and Wear Resistance

**Figure 5:**
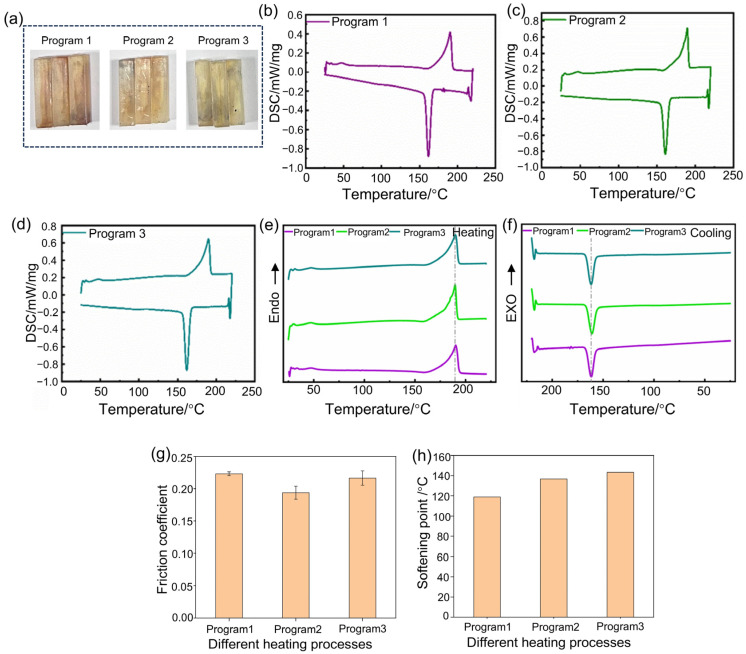
Effect of different heating programs on the crystallinity and wear resistance of Nylon 11. (**a**) Digital photos, (**b**–**d**) DSC curves of heating program 1, heating program 2, heating program 3, (**e**) heating curves, (**f**) cooling curves, (**g**) friction coefficient, (**h**) softening point.

Figure 5a presents digital photos of Nylon 11 wear-resistant specimens subjected to different heating programs. The samples appear slightly yellow, and no significant differences in appearance are observed among the three programmed heating programs. However, with an increasing number of holding cycles, the coloration of the specimens gradually deepens. Figure 5b–d displays the DSC curves of Nylon 11 subjected to different heating programs. Despite variations in the number of holding cycles, all curves exhibit distinct melting and crystallization peaks, indicating that the crystallinity of Nylon 11 remains intact regardless of the employed heating protocols.

The DSC curves for the heating and cooling processes are shown in Figure 5e,f. During the programmed heating process, multiple hold temperature periods did not have an impact on the melting behavior of Nylon 11, with the melting peak remaining consistently positioned between 189 °C and 190 °C. However, in the cooling curves, a slight shift in the crystallization peak of Nylon 11 is observed. This may be attributed to the ability of programmed heating to adequately extend the molecular chains of Nylon 11, resulting in crystallization at lower temperatures during the cooling process and corresponding higher crystallinity.

The melting point, melting enthalpy, and crystallinity of Nylon 11 of different heating programs are summarized in Table 3. After undergoing two holding cycles (maintained at 165 °C and 185 °C for 10 min each), the crystallinity of Nylon 11 increased, and excessive holding cycles did not further enhance its crystallization performance.

The friction coefficients are illustrated in Figure 5g. The Nylon 11 samples subjected to two holding temperature periods exhibited the lowest friction coefficient (friction coefficient: ~0.17), following the same trend as the DSC characterization results. This sample demonstrated the highest crystallinity, resulting in the lowest friction coefficient and superior wear resistance. Similarly, excessive holding temperature periods did not further enhance its wear resistance.

Figure 5h depicts the softening points of Nylon 11 samples subjected to different heating programs. The softening point increases with an increasing number of holding temperature periods. This phenomenon may be attributed to the extended time provided for molecular chain motion, which facilitates the orderly arrangement of molecular chains, ultimately leading to an elevation in the softening point of Nylon 11.

### 3.4. The Effect of the Varying Types and Concentrations of Nucleating Agents on the Crystallinity and Wear Resistance

**Figure 6:**
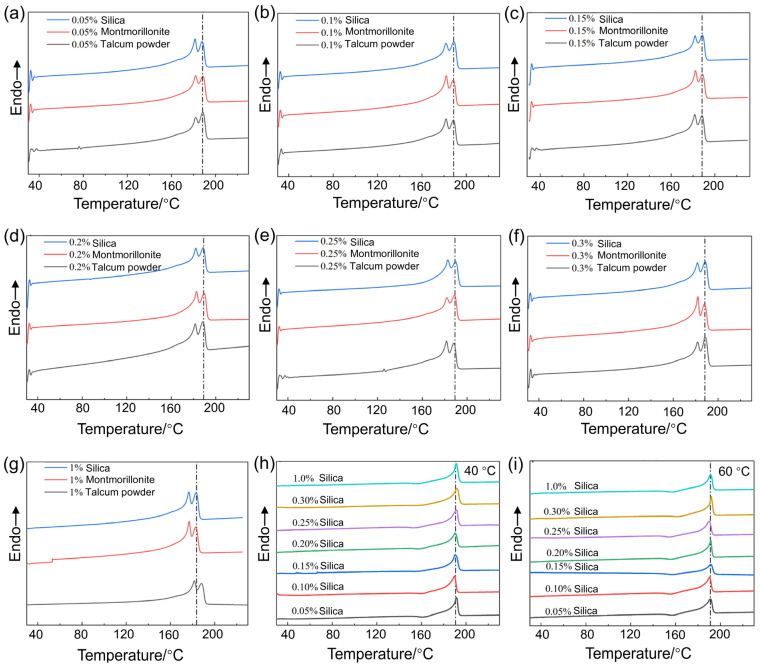
DSC curves of Nylon 11 with varying types and concentrations of nucleating agents (silica, talcum powder, and montmorillonite). (**a**) 0.05 wt%, (**b**) 0.1 wt%, (**c**) 0.15 wt%, (**d**) 0.2 wt%, (**e**) 0.25 wt%, (**f**) 0.3 wt%, (**g**) 1 wt%, (**h**) DSC curves of Nylon 11 crystallized at 40 °C using silica as a nucleating agent, (**i**) DSC curve of Nylon 11 crystallized at 60 °C using silica as a nucleating agent.

Figure 6a–g presents the DSC curves of Nylon 11 (crystallized at room temperature (25 °C)) with varying types and concentrations of nucleating agents. At the same concentration, samples using silica and talcum powder as nucleating agents exhibited similar T_m_, while samples with montmorillonite as the nucleating agent showed a slightly higher T_m_, although the difference was minor (less than 3 °C). When the nucleating agent was added at mass fractions of 0.05%, 0.1%, 0.15%, 0.2%, 0.25%, and 0.3%, the T_m_ of the samples remained relatively constant at approximately 189 °C. However, when the nucleating agent concentration abruptly increased to 1%, the T_m_ decreased to around 183 °C. In addition, the DSC curves reveal distinct shoulder peaks. During the heating, a notable cold crystallization exotherm is evident, indicating that the defect-laden crystals melt first, followed by a subsequent re-crystallization process. As the crystallization completes, the exothermic heat released surpasses the endothermic heat of melting, causing the heat flow curve to rise, which in turn forms the observed shoulder in the DSC curve.

Melting enthalpy and crystallinity of Nylon 11 with varying types and concentrations of nucleating agents at room temperature (25 °C) are summarized in Table 4. The crystallinity of Nylon 11 matrix materials varies with different mass fractions and types of nucleating agents. When montmorillonite was utilized as the nucleating agent, the crystallinity of Nylon 11 reached its peak value of 45.48% at a loading of 0.25 wt%, whereas the lowest crystallinity of 35.21% was obtained at a concentration of 1%. For talcum powder as the nucleating agent, the maximum crystallinity of 46.4% was observed at a content of 0.3%. When the concentration reached 1%, the crystallinity declined to 37.95%. In the case of silica as the nucleating agent, the highest crystallinity of 46.48% was obtained at a loading of 0.1%. With a concentration of 1%, the crystallinity decreased to 37.63%. Compared to pure Nylon 11 (crystallinity: 35.42%), the incorporation of nucleating agents leads to an increase in crystallinity for all samples. When the type of nucleating agent remains constant, the crystallinity first increases and then decreases with increasing mass fractions, exhibiting a similar trend across the three types of nucleating agents.

Given that the crystallinity of Nylon is highest when silica (0.1 wt%) is used as the nucleating agent, the crystallinity of Nylon 11 with silica as the nucleating agent at different crystallization temperatures were investigated. Figure 6h,i present the corresponding DSC curves of 40 °C and 60 °C, respectively. With the increasing mass fraction of the nucleating agent, the melting temperature of the samples exhibited no significant changes, remaining within the range of 190 °C to 191 °C. The melting enthalpy and crystallinity of Nylon 11 crystallized at 40 °C and 60 °C, using silica as a nucleating agent were summarized in Table 5 and Table 6, respectively. At a crystallization temperature of 40 °C, the variation in crystallinity still exhibited a trend of first increasing and then decreasing; however, the changes were relatively minor, remaining mostly within the range of 26% to 30%. When the crystallization temperature was raised to 60 °C, the trend in crystallinity was consistent with that observed at 40 °C, and the levels of crystallinity were similar as well. Nonetheless, the crystallinity values for both temperatures were significantly lower than those of Nylon crystallized at room temperature.

**Figure 7:**
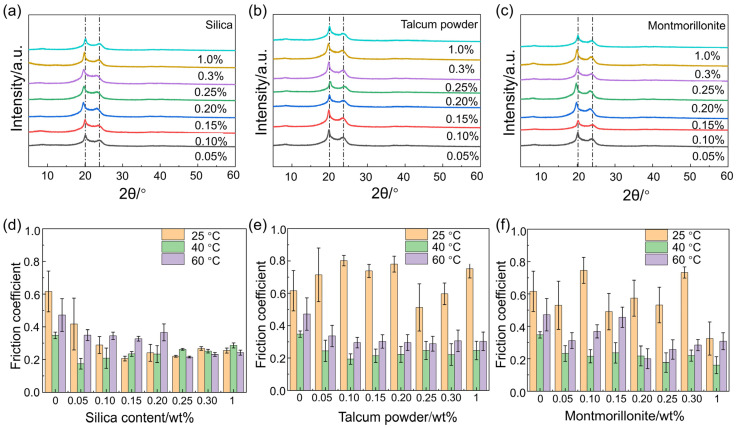
The XRD curves of Nylon 11 incorporated with different nucleating agents and the friction coefficients of Nylon 11 at various crystallization temperatures. (**a**–**c**) XRD curves of (**a**) silica, (**b**) talcum powder, (**c**) montmorillonite; (**d**–**f**) friction coefficients of (**d**) silica, (**e**) talcum powder, (**f**) montmorillonite.

Figure 7a–c is the XRD curves of Nylon 11 incorporating various types and concentrations of nucleating agents. Figure 7a displays the XRD curves of Nylon 11 incorporating varying mass fractions of silica. The results indicate that each spectrum features two prominent diffraction peaks at approximately 2θ = 20.1° and 2θ = 23.0°, suggesting that the crystallinity of the samples corresponds to the triclinic α-form. This form is characterized by amide groups positioned in a plane inclined to the chain axis, forming a triclinic unit cell with a structure similar to the hydrogen-bonded layered arrangement of PA66, wherein hydrogen bonds are established between parallel chains. The appearance of the two strong reflections in the XRD pattern results from the predominant role of hydrogen bonds in the crystalline structure of polyamides. The hydrogen-bonded layers are a key feature of this structure. The peak at approximately 2θ = 20.1° corresponds to the (100) plane of this crystal form, while the peak at around 2θ = 23.0° represents the overlaying (010) and (110) planes. The interplanar spacings associated with these strong reflections are approximately 0.44 nm and 0.37 nm, respectively, representing the distance between layers and the chain-to-chain distance projected within the layers. Additionally, the positions of the strong diffraction peaks in the XRD curves of all samples are consistent, occurring at approximately 2θ = 20.1° and 2θ = 23.0°. This indicates that no new crystal forms of Nylon 11 are present, demonstrating that the incorporation of different nucleating agents does not lead to a change in the crystalline form of Nylon 11. Furthermore, the absence of characteristic diffraction peaks from the various nucleating agents in the XRD patterns suggests that these agents mix uniformly within the Nylon 11 matrix [25].

Figure 7d–f are the friction coefficients of Nylon 11 incorporated with different nucleating agents at various crystallization temperatures. The results indicate that the type and content of the nucleating agents, as well as the crystallization temperature, significantly influence the frictional properties of Nylon 11. When silica is used as the nucleating agent, the friction coefficient gradually decreases with increasing mass fraction of the nucleating agent. And the coefficient of friction tends to be stabilized when the silica content reaches 0.1%, and changes in temperature and content have little effect on it, as seen in Figure 7d. However, when talcum powder or montmorillonite is used as the nucleating agent, the friction coefficients of samples crystallized at 40 °C and 60 °C are significantly lower than those of samples crystallized at 25 °C, and the lowest friction coefficient is observed at a crystallization temperature of 40 °C, as depicted in Figure 7e,f. And the friction coefficient initially decreases and then stabilizes, as the amount of nucleating agent increases. This result indicates that nylon 11 with silica as a nucleating agent has better friction stability compared to montmorillonite and talcum powder.

## 4. Conclusions

In conclusion, the crystallinity behavior and wear resistance of Nylon 11 can be affected by the processing methods and the nucleating agents of the Nylon. The crystalline phase formed by Nylon 11 is the α-phase, which exhibits good stability. Enhanced wear resistance is observed when the pre-treatment temperature is maintained between 80 °C and 100 °C, the melting temperature exceeds 195 °C, and the friction coefficient of Nylon at this point is ~0.17. Additionally, a proper heating program can also decrease the friction coefficient. Among the inorganic nucleating agents, silica enables Nylon 11 to have higher crystallinity (46.48%) and better friction stability; Nylon 11 demonstrates the lowest coefficient of friction at a crystallization temperature of 40 °C.

## Figures and Tables

**Table 1:** Melting point, melting enthalpy, and crystallinity of Nylon 11 at different pre-treatment temperatures.

Pre-Treatment Temperature/°C	T_m_/°C	H_m_/J·g^−1^	X_c_/%
Untreated	189.90	58.39	30.89
60	189.46	57.87	30.61
80	190.36	51.61	27.31
100	190.00	64.83	34.30
120	189.97	70.61	37.36
140	190.03	69.89	36.98
160	189.73	67.07	35.49
180	189.73	52.71	27.89

T_m_: melting point; H_m_: melting enthalpy; X_c_: crystallinity.

**Table 2:** Melting point, melting enthalpy, and crystallinity of Nylon 11 at different melting temperatures.

Melting Temperature/°C	T_m_/°C	H_m_/J·g^−1^	X_c_/%
190	191.30	58.86	31.14
195	189.36	65.52	34.67
200	190.62	58.58	31.00
205	188.74	63.74	33.72
210	189.91	60.65	32.09

**Table 3:** Melting point, melting enthalpy, and crystallinity of Nylon 11 of different heating programs.

Sample	T_m_/°C	H_m_/J·g^−1^	X_c_/%
Program 1	190.10	56.46	29.87
Program 2	189.60	64.44	34.10
Program 3	189.80	59.90	31.69

**Table 4:** Melting enthalpy, and crystallinity of Nylon 11 crystallized at room temperature (25 °C) using different nucleating agents.

Content/wt%	Montmorillonite		Talcum Powder		Silica	
	ΔH_m_/J·g^−1^	X_c_/%	ΔH_m_/J·g^−1^	X_c_/%	ΔH_m_/J·g^−1^	X_c_/%
0	~	~	~	~	66.95	35.42
0.05	68.96	36.51	81.99	43.40	80.89	42.82
0.1	83.65	44.34	86.04	45.76	87.55	46.48
0.15	84.19	44.61	79.71	42.17	78.55	41.56
0.2	85.75	45.46	84.19	44.54	79.86	42.25
0.25	85.75	45.48	77.61	41.06	79.92	42.29
0.3	77.33	41.04	87.43	46.40	82.31	43.68
1	65.88	35.21	71.01	37.95	69.91	37.63

Red data represent the maximum crystallinity value.

**Table 5:** The melting enthalpy, and crystallinity of Nylon 11 crystallized at 40 °C.

Silica Content/wt%	ΔH_m_/J·g^−1^	X_c_/%
0.05%	50.18	26.55
0.10%	51.44	27.22
0.15%	53.06	28.07
0.20%	48.17	25.49
0.25%	53.11	28.10
0.30%	57.76	30.56
1.0%	55.71	29.48

**Table 6:** The melting enthalpy, and crystallinity of Nylon 11 crystallized at 60 °C.

Silica Content/wt%	ΔH_m_/J·g^−1^	X_c_/%
0.05%	54.64	28.91
0.10%	54.92	29.06
0.15%	39.95	21.14
0.20%	53.08	28.08
0.25%	52.00	27.51
0.30%	51.64	27.32
1.0%	54.42	28.79

## Data Availability

No new data were created or analyzed in this study. Data sharing is not applicable to this article.
